# Multi-centered clinical validation demonstrating superior precision in lupus diagnosis: T cell autoantibodies and TC4d outperform conventional lupus erythematosus biomarkers

**DOI:** 10.3389/fimmu.2025.1518208

**Published:** 2025-02-26

**Authors:** Vasileios Kyttaris, Daniel J. Wallace, Arezou Khosroshahi, Andrew Concoff, Nicole Wilson, Chau Ching Liu, Susan Manzi, Joseph Ahearn, Sepehr Taghavi, Touba Warsi, Stanley Park, Christine Schleif, Brittany D. Partain, Tyler O’Malley

**Affiliations:** ^1^ Beth Israel Deaconess Medical Center, MD, Boston, MA, United States; ^2^ Cedars-Sinai Medical Center, Los Angeles, CA, United States; ^3^ Emory University, Atlanta, GA, United States; ^4^ Exagen, Vista, CA, United States; ^5^ Allegheny Health Network, Pittsburgh, PA, United States

**Keywords:** T cell biology, lymphocyte autoantibodies, complement activation in SLE, SLE biomarkers, diagnostic biomarkers

## Abstract

**Introduction:**

T Cell autoantibodies, TIgG and TIgM, as well as the T Cell-bound complement protein fragment C4d (TC4d) are novel diagnostic biomarkers that have demonstrated high specificity and sensitivity for SLE. The present study aims to characterize the clinical performance characteristics of the emergent T Cell biomarkers in a multi-center clinical validation cohort.

**Methods:**

A cohort of 400 adult patients enrolled across 3 academic and 2 community-based autoimmune rheumatic centers, comprised of 105 SLE patients, 173 patients with autoimmune rheumatic diseases (ARD), 83 apparently healthy volunteers (AHV) and 39 other (non-autoimmune) disease (OD) controls were tested for TC4d, TIgG, TIgM and an extensive autoantibody profile. Diagnostic specificity was assessed against the ARD, AHV and OD groups, individually. Semi-quantitative flow cytometry analysis included TIgG and TIgM autoantibodies, cell-bound complement activation products (CB-CAPs), TC4d, erythrocyte-bound C4d (EC4d) and B lymphocyte-bound C4d (BC4d). Conventional autoantibodies and soluble complement proteins, C3 and C4, were assessed by ELISA and immunoturbidimetry, respectively.

**Results:**

ROC analysis distinguishing ANA-positive (ANA+) SLE (N = 91) from ARD, TIgG, BC4d and TC4d demonstrated AUC values 0.81, 0.80 and 0.79, respectively, outperforming anti-dsDNA (0.72), C3 (0.69), TIgM (0.67), C4 (0.66) and anti-Smith (0.61). A similar ranking of discriminatory power was observed in ROC analysis distinguishing ANA+ SLE vs. OD as well as ANA+ SLE vs. AHV. At 95% diagnostic specificity for SLE vs. AHV, the sensitivity (95% CI) of TC4d, TIgG and TIgM for SLE was 58.1% (48.1 – 67.7%), 31.4% (22.7 – 41.2%) and 29.5% (21.0 – 39.2%), respectively. The T Cell SLE biomarkers uniquely identified 19% (20/105) of SLE patients who were otherwise negative (serologically inactive) for conventional SLE autoantibodies and had normal serum complement levels. Among the serologically inactive SLE subset, the T Cell SLE biomarkers collectively identified 53% of subjects.

**Conclusions:**

The novel SLE biomarkers TC4d, TIgG and TIgM consistently outperform conventional markers across multiple cohorts. Their integration enhances diagnostic sensitivity, especially in SLE-specific autoantibody negative patients with normal complement levels. When coupled with conventional biomarkers, these novel tests may enable earlier and more accurate SLE detection, leading to more timely diagnosis and treatment.

## Introduction

Systemic Lupus Erythematosus (SLE) is a chronic autoimmune disease with protean manifestations characterized by a loss of tolerance in innate and adaptive immunity (1). Among the putative mechanisms leading to the pathogenic breakdown of immune tolerance in SLE is the development of autoreactive T Cells, which contribute to pathologic activation of B cells, dysfunction of regulatory T Cells and aberrant production of pro-inflammatory cytokines (2, 3).

Efforts to understand the drivers of T Cell dysfunction in SLE have examined anti-lymphocyte autoantibodies (ALAs), IgG and IgM, which have diverse specificities for CD3, CD4, CD45 and IL-2R (4–7). Anti-T Cell IgM (TIgM) antibodies may contribute to SLE pathogenesis through activation of the classical complement pathway, as IgM antibodies have 500-fold greater potency in activating complement compared to IgG (8, 9). Autoantibodies targeting the T Cell Receptor (TCR)/CD3 have been demonstrated to activate Ca^2+^/calmodulin-dependent kinase IV (CaMKIV), resulting in diminished IL-2 production and low serum IL-2 levels are commonly observed in SLE (6). Additionally, the function of the CD45 Protein Tyrosine Phosphatase (PTP) enzyme is diminished in a portion of SLE patients, driven at least in part by inhibitory anti-CD45 directed autoantibodies (10). Given the central roles that T Cell dysregulation and classical complement activation play in SLE pathogenesis, it follows that biological signatures of these phenomena may represent unique diagnostic biomarkers of disease.

Two recent single-center studies (11, 12) in the U.S. and China have characterized the diagnostic sensitivity and specificity of complement protein 4 derived ligand (C4d) bound to CD3+ T Cells (TC4d) in combination with TIgG and TIgM, compared to conventional autoantibodies (i.e., anti-dsDNA, anti-Smith) and serum complement proteins (i.e., C3 and C4) traditionally used in the diagnosis of SLE. In each study, exposure to SLE sera exclusively exhibiting TIgG, TIgM and TC4d signatures resulted in classical complement activation and transfer of TC4d signatures to normal T lymphocytes. Collectively, these findings underscore the potential of T Cell biomarkers to address the significant unmet need for sensitive and specific biomarkers of SLE (13), particularly where conventional antibody testing is poorly sensitive (14).

SLE diagnosis is confounded by a variety of factors, including heterogeneous and non-specific signs and symptoms, as well as conventional biomarkers that lack diagnostic sensitivity, particularly in early disease (15). Consequently, the time from symptom onset to diagnosis is considerable, as observed in multiple longitudinal lupus cohorts (16–18). In the U.S., the average time from symptom onset to diagnosis is nearly 6 years, with 63% of lupus patients self-reporting at least one misdiagnosis before their SLE diagnosis (16). Similarly, a German lupus cohort reported an average of 5 years from symptom onset to diagnosis and demonstrated a significant direct relationship between time to diagnosis and increased disease activity, disease-related damage and fatigue (17). Concordant with findings in Germany and the U.S, a Greek lupus cohort reported a median 2-year delay from symptom onset to diagnosis with nearly 56% experiencing delays of at least 12 months and the majority consulting three different physicians before receiving a formal diagnosis (18).

The impact of SLE diagnosis can be understood through a combination of longitudinal inception cohorts and retrospective commercial claims analyses that demonstrate the protective effects associated with prompt and appropriate treatment intervention. A retrospective U.S. commercial claims analysis of SLE patients found that early diagnosis (within 6 months of symptom onset) was associated with lower rates of mild, moderate and severe flares, in addition to decreased rates of hospitalization compared to SLE patients experiencing a delayed diagnosis (19). Moreover, in another study, uncontrolled SLE disease activity was closely associated with disease progression, characterized by the accumulation of irreversible organ damage, which itself portends future damage (20). The pervasiveness of diagnostic delay and its impact on patient quality of life has been reflected in the findings of multiple longitudinal SLE cohorts wherein 33-50% of SLE patients sustain irreversible organ damage within 5 years of diagnosis (21–23). Lastly, a recent study (24) found that the rate of incident permanent damage in SLE patients was highest in the first year after diagnosis (20% of patients experiencing damage) and progressively declined over the subsequent six years. Thus, the collective data underscore a finite window of opportunity early in the SLE disease course where appropriate diagnosis and therapeutic intervention hold the greatest potential to affect the trajectory and impact of the disease.

The gold standard for diagnosing SLE remains the gestalt impression of a well-trained clinician, informed by factors including demographics, presenting signs and symptoms, and available biomarkers (25). Conventional SLE biomarkers including antinuclear antibodies (ANA), anti-dsDNA and complement C3 and C4 represent cornerstones in SLE diagnosis and have been included in classification criteria for SLE. However, the low specificity/high sensitivity of ANA and low sensitivity/high specificity of the remainder of these biomarkers for SLE, can contribute to delays in an accurate diagnosis (26–28). Therefore, by employing biomarkers with limited test performance, the traditional approach bears recognized limitations, contributes to misdiagnosis and results in detrimental diagnostic delay.

Cell-bound complement activation products (CB-CAPs) offer promise in addressing these challenges. CB-CAPs, such as C4d, form covalent bonds that deposit irreversibly on the cell membranes of various cells including erythrocytes (EC4d), B-cells (BC4d) and T-cells (TC4d). Cell-bound C4d reflects a sustained measure of complement activation, in contrast to soluble complement markers including C3 and C4, which are more transiently variable (29). Prior studies have demonstrated that CB-CAPs outperform C3 and C4 in distinguishing SLE patients, with 22% higher sensitivity and comparable specificity (30). Further, the combination of CB-CAPs with conventional SLE and CTD markers in a validated 2-tier diagnostic algorithm (31), resulted in both 20-25% higher positive predictive value for SLE diagnosis and initiation of SLE-related medications, potentially shortening the time to diagnosis and treatment and thereby improving patient outcomes (32).

### Study objective

The present study aims to demonstrate the clinical validity of novel T Cell SLE biomarkers, TIgG and TIgM, as well as TC4d in comparison to conventional biomarkers of SLE in a diverse multi-center clinical validation cohort consisting of both academic and community-based rheumatology practices.

## Methods

### Study design

A cohort of 400 adult subjects were consented, in compliance with the Helsinki Declaration, and enrolled at 3 academic and 2 community autoimmune rheumatic disease centers. Institutional or central IRB review of the study protocol was conducted at each investigative site prior to patient enrollment. The study cohort was inclusive of 105 SLE patients meeting the 1997 ACR classification criteria for SLE, 173 patients with autoimmune rheumatic diseases (ARD), 39 other (non-autoimmune) other disease controls (OD) and 83 apparently healthy volunteers (AHV). In the ARD cohort, RA subjects were required to meet 2010 ACR classification criteria for RA. Other disease controls were identified based on ICD9/10 diagnostic codes recorded in the patient’s medical record.

### Biomarker assay methodology

Measurement of cell-bound complement activation products, erythrocyte-bound C4d (EC4d) and B lymphocyte-bound C4d (BC4d) was performed by semi-quantitative flow cytometry assays as previously described (31). Anti-Smith IgG, anti-U1-RNP IgG, anti-RNP70 IgG, anti-Ro60 IgG and anti-Ro52 IgG assays were performed by enzyme-linked fluorescent-enzyme immunoassays (ELISA, ThermoFisher, Uppsala, Sweden). Anti-dsDNA IgG, anti-C1q IgG and ANA IgG were measured by ELISA (Werfen, San Diego, CA). ANA by indirect immunofluorescence was assessed using HEp-2 cells with a positive threshold based on a serum titer of ≥ 1:80 (Werfen, San Diego, CA). Complement C3 and C4 levels were determined using immunoturbidimetry (The Binding Site, San Diego, CA).

The detailed protocol for the measurement of TC4d, TIgG and TIgM levels by semi-quantitative flow cytometry as described in the **Supplementary Methods**. Briefly, lymphocytes are isolated, washed and stained with a cocktail containing mouse monoclonal antibodies against human C4d (specific antibody), human IgG and IgM, or the appropriate non-specific antibody (isotype). The antibody cocktail includes an anti-CD3 antibody for T Cell identification. The mean fluorescence intensity (MFI) of the isotype background control and target (C4d, IgG, IgM) is collected from every specimen, and the net MFI is determined by the difference between specific and isotype MFI. All flow cytometry analysis was performed on a Lyric (BD, San Diego, CA) with a 3-laser configuration.

### Data collection and processing

Natural log transformation was applied for the visualization and presentation of biomarker values, after replacing negative values with 0 and adding the minimum value greater than 0 for each biomarker to all its corresponding values.

### Statistical methodology

Analyses were conducted using R (2024.04.1 + 748 “Chocolate Cosmos” Release for windows) and Python 3.12.2. Descriptive statistics with 95% confidence intervals were calculated for diagnostic performance characteristics, including sensitivity, specificity, positive likelihood ratio (+LR), negative likelihood ratio (-LR), odds ratios, positive predictive value (PPV), negative predictive value (NPV) and Youden’s index (sensitivity + specificity -1). All reported adjusted p-values were obtained using the Benjamini-Hochberg procedure to control for the false discovery rate arising from multiple comparisons. McNemar’s test was utilized to determine the statistical significance of differences in clinical performance characteristics. The Youden’s index reflects a biomarker performance statistic with values between 0 to 1 taking into account the overall sensitivity and specificity of a test where a perfect test would have a score of 1. Receiver operating characteristic (ROC) curves and area under the curve (AUC) were analyzed for each biomarker stratified by ANA status with different control groups evaluated independently. DeLong’s statistical test was employed to compare differences in AUCs among diagnostic biomarkers with statistical significance defined as p < 0.05 for all comparisons.

Correlation between biomarkers and clinical features was determined using non-parametric Spearman’s rank test or Fisher’s exact test as appropriate. Differences in continuous variables stratified by biomarker positive/negative status were evaluated using the Mann Whitney U-test, t-test, or Kruskal Wallis tests as applicable with Wilcoxon rank sum test used for effect size. For anomaly detection, an ensemble of models was utilized to evaluate potential outliers among the samples for the biomarkers. The “Isolation Forest with Calibration” model demonstrated superior performance in identifying anomalies, and the resulting anomaly scores were subsequently incorporated into further statistical analyses to investigate potential common causes of outlier status across all biomarkers.

## Results

### Demographic characteristics

Demographic characteristics and ANA status are detailed in **Table 1**. The SLE cohort was predominantly White (75%) and female (95%), with a mean (SD) age of 50.8 (± 13.5) years old. ANA by ELISA and/or IFA was positive in 87% of SLE subjects. AHV subjects were also majority White (51%) and female (54%), with a mean (SD) age of 41.9 (± 15.1). ARD subjects consisted mostly of RA (N = 82), Sjögren Disease (N = 29) and a collection of other diseases (N = 71) enumerated in the footer of **Table 1**. The ARD group was predominantly White (78-94%) and female (61-97%) with a mean (SD) age ranging from 55.1 (± 12.4) to 57.2 (± 14.8) years old across individual disease groups. Finally, the non-autoimmune, OD, group was predominantly White (82%) and female (79%), with a mean (SD) age of 51.8 (± 15.5) years.

**Table 1:** Demographic characteristics and ANA status at the time of blood collection.

					Autoimmune Rheumatic Disease (ARD)		
	Total	SLE	AHV*	OD**	RA	Other***	Sjögren
	(N=400)	(N=105)	(N=83)	(N=39)	(N=82)	(N=62)	(N=29)
Ethnicity							
White	299 (75%)	79 (75%)	42 (51%)	32 (82%)	64 (78%)	58 (94%)	24 (83%)
Hispanic	31 (8%)	4 (4%)	14 (17%)	5 (13%)	5 (6%)	0 (0%)	3 (10%)
Black	36 (9%)	15 (14%)	5 (6%)	2 (5%)	11 (13%)	2 (3%)	1 (3%)
Asian	20 (5%)	3 (3%)	16 (19%)	0 (0%)	1 (1%)	0 (0%)	0 (0%)
Native American	0 (0%)	0 (0%)	0 (0%)	0 (0%)	0 (0%)	0 (0%)	0 (0%)
Other	5 (1%)	1 (1%)	3 (4%)	0 (0%)	1 (1%)	0 (0%)	0 (0%)
Not Disclosed	9 (2%)	3 (3%)	3 (4%)	0 (0%)	0 (0%)	2 (3%)	1 (3%)
Subject is Female	299 (75%)	95 (90%)	45 (54%)	31 (79%)	62 (76%)	38 (61%)	28 (97%)
Age at Visit – Mean (SD)	51.4 (± 14.9)	50.8 (± 13.5)	41.9 (± 15.1)	51.8 (± 15.5)	57.2 (± 14.8)	55.6 (± 11.5)	55.1 (± 12.4)
ANA By Method Result n(%)							
Positive by Both Methods	147 (37%)	78 (74%)	4 (5%)	6 (15%)	23 (28%)	15 (24%)	21 (72%)
Negative by Both Methods	150 (38%)	14 (13%)	60 (72%)	21 (54%)	23 (28%)	31 (50%)	1 (3%)
Only Positive by ELISA	67 (17%)	11 (10%)	14 (17%)	8 (21%)	22 (27%)	7 (11%)	5 (17%)
Only Positive by Hep2	36 (9%)	2 (2%)	5 (6%)	4 (10%)	14 (17%)	9 (15%)	2 (7%)

*AHV - apparently healthy volunteers who consented to provide blood for biomarker discovery and/or clinical validation.

**OD - other rheumatic diseases that are non-autoimmune in nature: Fibromyalgia [n=14]; Chronic Localized Pain [n=13]; Osteoarthritis [n=7]; Gout [n=2]; Undifferentiated Arthritis [n=2]; Osteoporosis [n=1].

***Other ARD - other rheumatic diseases that are autoimmune in nature: Psoriatic Arthritis [n=24]; Spondyloarthropathy [n=8]; Non-Rheumatoid Inflammatory Arthritis [n=7]; Polymyalgia Rheumatica [n=4]; Sarcoidosis [n=3]; Systemic Sclerosis [n=3]; Adult-Onset Still’s Disease [n=2]; Myositis [n=2]; Still’s Disease [n=2]; Axial Spondyloarthritis [n=1]; Dermatomyositis [n=1]; Enteropathic Arthropathy [n=1]; Idiopathic Inflammatory Myopathy [n=1]; Non-radiographic Axial Spondyloarthritis [n=1]; Psoriasis [n=1]; Wegeners Granulomatosis [n=1].

Medication use in this cohort (all dosages in milligrams) was as follows: of the 105 SLE subjects, 81% were treated with hydroxychloroquine (mean dose = 223.5, SD = 57), 40% percent with oral corticosteroids (5.5, 4), 30% with mycophenolate (916.7, 376.4), 19% with belimumab (217.2, 114.6), 10% with methotrexate (6.6, 7.3), 9% with azathioprine (86.1, 69.7), and 1 subject was taking cyclophosphamide (dose = 500) (**Table 2**). We performed separate logistic regression analyses to evaluate whether each of the biomarkers included in the analysis were predictive of the use of these medications. Each regression model included all markers as independent variables and the use of a specific medication as the binary outcome. The data are not significant predictors of medication use in this cohort (data not shown).

**Table 2:** Medication table for SLE subjects (N = 105) at the time of blood draw.

SLE Subject Medication Data		
Medication	Percent	Mean Dosage, mg (SD)
Hydroxychloroquine	81%	223.5 (57)
Prednisone	40%	5.5 (4)
Mycophenolate	30%	916.7 (376.4)
Belimumab	19%	217.2 (114.6)
Methotrexate	10%	6.6 (7.3)
Azathioprine	9%	86.1 (69.7)
Cyclophosphamide*	1%	500 (N/A)

Mean and standard deviation (SD) dosage prescribed at the time of blood collection. * Only one patient was taking Cyclophosphamide, thus a standard deviation cannot be calculated for this dosage.

**Figure 1:**
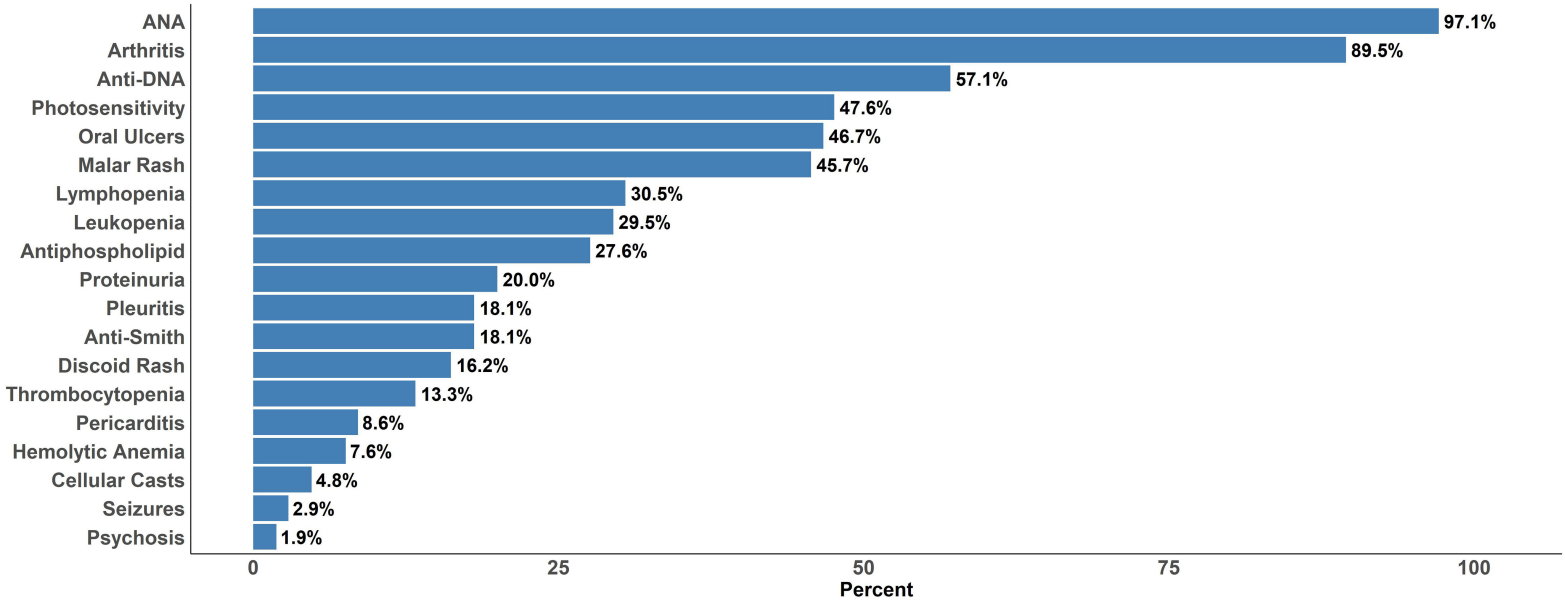
Fulfillment of 1997 ACR Classification Criteria for SLE based on cumulative (historical) fulfillment among the 105 SLE subjects included in the analysis.

The majority of the SLE cohort met the following classification criteria (**Figure 1**), each assessed historically: ANA (97.1%), arthritis (89.5%), anti-dsDNA (57.1%) and photosensitivity (47.6%). Hematological sub-criteria fulfillment was less prevalent with lymphopenia (30.5%) being most common, followed by leukopenia (29.5%), thrombocytopenia (13.3%) and hemolytic anemia (7.6%). Skin and mucosal manifestations, including oral ulcers, malar rash and discoid rash criteria were fulfilled in 46.7%, 45.7% and 16.2%, respectively. Renal criteria, including proteinuria and cellular casts were met in 20.0% and 4.8%, respectively. The mean (± SD) SLEDAI-2K score and non-serological (ns) SELENA-SLEDAI-2K score at the visit where blood was drawn for testing was 3.48 (± 4.22) and 2.43 (± 3.95), respectively. In general, clinical criteria made up the bulk of SLE disease activity at the time of testing while the majority of SLE patients had met immunological criteria.

### ROC analysis: ANA positive (ANA+) SLE vs. controls

**Figure 2:**
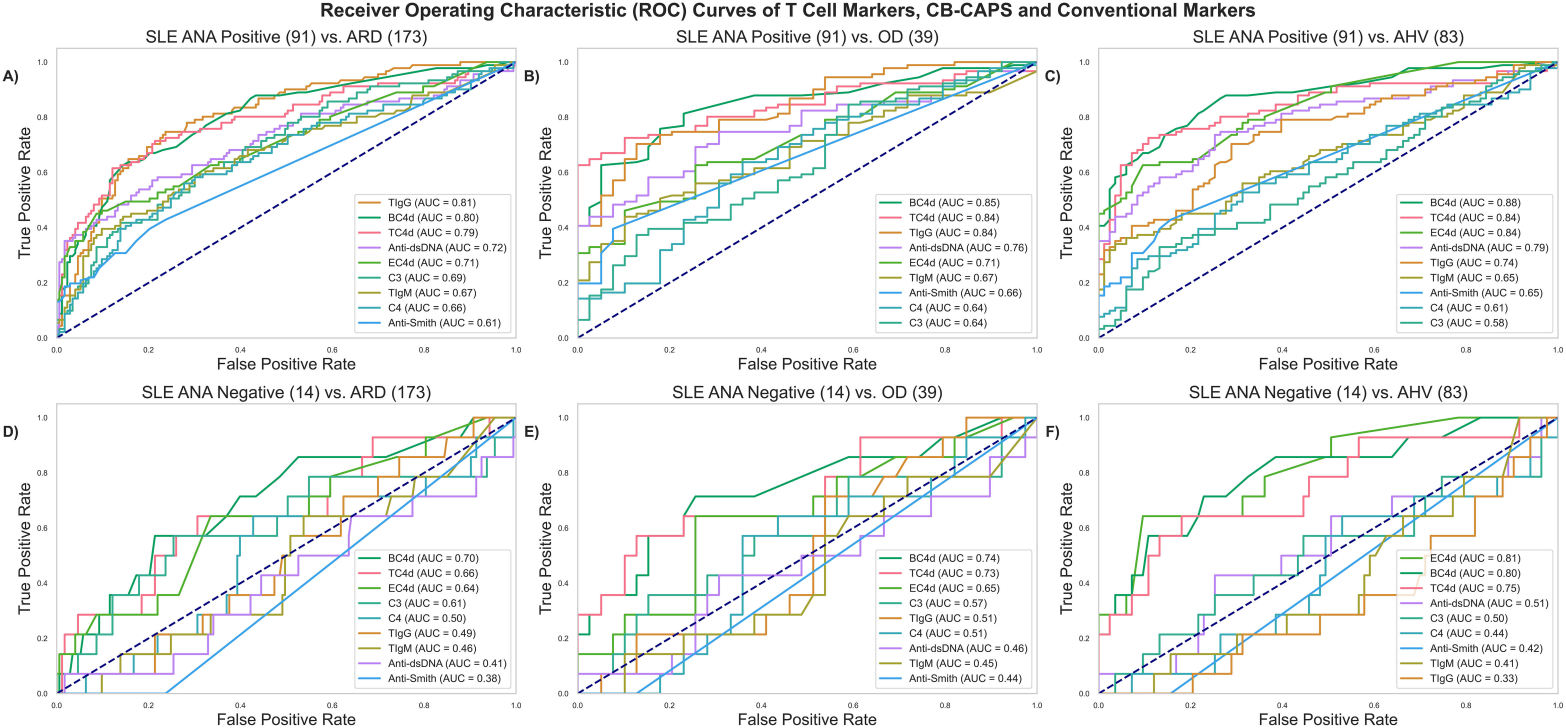
ROC analyses comparing T Cell SLE biomarkers (TC4d, TIgG and TIgM) to CB-CAPs (EC4d and BC4d) and conventional SLE biomarkers. ANA positive (ANA+) SLE is defined as SLE patients with a positive result by either ELISA solid phase assay and/or IFA (positive threshold based on a serum titer ≥ 1:80). **(A)** ANA+ SLE vs. ARD **(B)** ANA+ SLE vs. OD **(C)** ANA+ SLE vs. AHV **(D)** ANA- SLE vs. ARD **(E)** ANA- SLE vs. OD **(F)** ANA- SLE vs. AHV.

**Figure 2A** compares biomarker performances in ANA-positive (ANA+ by either method at the time of blood collection) SLE versus ARD patients, revealing that TIgG (AUC: 0.81) significantly outperforms anti-dsDNA (AUC: 0.72, p = 0.02), EC4d (0.71, p = 0.01), C3 (0.69, p < 0.01), C4 (0.66, p < 0.01) and anti-Smith (0.61, p < 0.01) but not BC4d (0.80, p = 0.84). TC4d (AUC: 0.79) significantly outperforms EC4d (p = 0.04), C3 (p < 0.01), C4 (p < 0.01) and anti-Smith (p <0.01) but not anti-dsDNA (p = 0.09) and BC4d (p = 0.59). TIgM (AUC: 0.67) was significantly outperformed by BC4d (p < 0.01), but outperformed C3 (p < 0.01) and C4 (p < 0.01). In the context of distinguishing ANA+ SLE vs. OD (**Figure 2B**), TC4d (AUC:0.84) significantly outperformed EC4d (0.71, p = 0.01), anti-Smith (0.66, p <0.01), C3 (0.64, p <0.01), C4 (0.64, p < 0.01) but not anti-dsDNA (0.76, p = 0.06) or BC4d (0.85, p = 0.89). TIgG (AUC: 0.84) demonstrated a similar set of significant differences as TC4d while TIgM (AUC: 0.67) was significantly worse than BC4d (p < 0.01) and significantly better than C3 (p < 0.01) and C4 (p < 0.01).

In the differentiation of SLE vs. AHV (**Figure 2C**), TC4d (AUC: 0.84) significantly outperformed anti-Smith (0.65, p < 0.01), C4 (0.61, p < 0.01) and C3 (0.58, p < 0.01) but not EC4d (0.84, p = 0.98), BC4d (0.88, p = 0.24) or anti-dsDNA (0.79, p = 0.19). TIgG (AUC: 0.74) significantly outperformed anti-Smith (p = 0.02), C4 (p < 0.01), C3 (p < 0.01) but was significantly outperformed by EC4d (p < 0.01), BC4d (p < 0.01). TIgG and anti-dsDNA were not significantly different (p = 0.32). TIgM (AUC: 0.65) significantly outperformed C4 (p <0.01) and C3 (p < 0.01) but was outperformed by EC4d (p<0.01), BC4d (p<0.01) and anti-dsDNA (p <0.01). TIgM and anti-Smith were not significantly different in performance (p = 0.88).

### ROC analysis ANA negative (ANA-) SLE vs. controls

Given the inherent challenges associated with the minority of SLE patients with transitory positive ANA results, a subset analysis was performed to characterize the diagnostic value of the T Cell biomarkers in this context. Among the SLE subjects, 13% (N = 14) tested negative by both ANA methods (ELISA and IFA) at the time of blood collection and were assessed using the same ROC analyses as applied to the ANA+ group, above. In differentiating ANA- SLE from ARD (**Figure 2D**), TC4d (AUC: 0.66) significantly outperformed C3 (0.61, p =0.04), anti-dsDNA (0.41 p <0.01), anti-Smith (0.38, p <0.01) while performances against all other biomarkers were not significantly different. TIgG and TIgM did not significantly outperform any of the conventional biomarkers. In an analysis of differentiating ANA- SLE from OD (**Figure 2E**), TC4d (AUC: 0.73) significantly outperformed C3 (0.57, p = 0.03), anti-dsDNA (0.46, p = 0.04) and anti-Smith (0.44, p < 0.01). TIgG (0.51) and TIgM (0.45) did not significantly outperform any other biomarker in this diagnostic context. Lastly, in the differential between ANA- SLE and AHV (**Figure 2F**), TC4d (AUC: 0.75) significantly outperformed anti-Smith (0.42, p < 0.01) and nearly reached significance for superior performance against anti-dsDNA (0.51, p = 0.06) and C3 (0.50, p = 0.05).

### T cell and conventional biomarker performance characteristics

Biomarker performance characteristics are summarized in **Table 3**. At 94% specificity for SLE versus AHV, TC4d had a sensitivity of 58.1% (95% CI: 48.1-67.7%), higher than anti-dsDNA (33.3%, 95% CI: 24.4-43.2%) and anti-Smith (11.4%, 95% CI: 6.0-19.1%). These differences were statistically significant, with a strong effect size assessed using McNemar’s test and Cohen’s *g* for effect size, as summarized in **Table 4**. The odds ratio for TC4d positive and anti-dsDNA negative versus TC4d negative and anti-dsDNA positive was 5.3 (95% CI: 2.4–14.2; adjusted p<0.001, *g*=0.7). Notably, since there were no cases where TC4d was negative and anti-Smith was positive, the odds ratio for TC4d positive and anti-Smith negative versus TC4d negative and anti-Smith positive was undefined (adjusted p<0.001, *g*=1). At similar specificity for SLE vs. AHV (94.0-95.2%), TIgG and TIgM sensitivity for SLE was 31.4% (95% CI: 22.7-41.2%) and 29.5% (95% CI: 21.0-39.2%), respectively. EC4d and BC4d were 100% specific for SLE versus AHV, with sensitivity for SLE of 16.2% (95% CI: 9.7-24.7%) and 31.4% (95% CI: 22.7-41.2%), respectively. Serum Complement protein levels (C3 and C4) exhibited poor sensitivity for SLE at 95-96% specificity for SLE vs. AHV, with sensitivity for SLE of 4.8% (95% CI: 1.6-10.8%) and 9.5% (95% CI: 4.7-16.8%), respectively. Additional extractable nuclear antigen (ENA) autoantibody data encompassing anti-Ro60, anti-Ro52, anti-U1-RNP, anti-RNP70 and anti-C1q is presented in **Supplementary Table 1**.

**Table 3:** Clinical Performance Characteristics of T Cell biomarkers (based on the 95^th^ percentile of AHV as the reference interval threshold), CB-CAPs and conventional SLE biomarkers, based on previously established diagnostic thresholds.

	Sensitivity % [95% CI]	Specificity % [95% CI]			SLE vs AHV [95% CI]					
Biomarker	SLE	AHV	ARD	OD	+LR	-LR	DOR	Youden’s index	PPV	NPV
TC4d	58.1 [48.1 - 67.7]	94.0 [86.5 – 98.0]	86.1 [80.1 - 90.9]	100 [91 - 100]	9.6 [3.9 - 23.8]	0.4 [0.3 - 0.6]	21.6 [8.1 - 57.8]	52.1 [38.6 - 65.5]	92.4 [83.2 - 97.5]	63.9 [54.7 - 72.4]
TIgG	31.4 [22.7 - 41.2]	94.0 [86.5 – 98.0]	92.5 [87.5 - 95.9]	100 [91 - 100]	5.2 [1.9 - 14.3]	0.7 [0.6 - 0.9]	7.1 [2.6 - 19.3]	25.4 [8.7 - 42.1]	86.8 [71.9 - 95.6]	52 [43.7 - 60.2]
TIgM	29.5 [21.0 - 39.2]	95.2 [88.1 - 98.7]	92.5 [87.5 - 95.9]	92.3 [79.1 - 98.4]	6.1 [2.0 - 18.8]	0.7 [0.6 - 0.9]	8.3 [2.8 - 24.6]	24.7 [8.0 - 41.4]	88.6 [73.3 - 96.8]	51.6 [43.4 - 59.8]
BC4d	31.4 [22.7 - 41.2]	100 [95.7 - 100]	95.4 [91.1 - 98]	100 [91 - 100]	13.7 [3.1 - 60.7]	0.7 [0.6 - 0.9]	76.1 [4.6 - 1264.4]	31.4 [15.6 - 47.3]	100 [89.4 - 100]	53.5 [45.4 - 61.6]
EC4d	16.2 [9.7 - 24.7]	100 [95.7 - 100]	99.4 [96.8 - 100]	100 [91 - 100]	7.0 [1.2 - 41.4]	0.9 [0.7 - 1.1]	32.1 [1.9 - 542.5]	16.2 [-1.3 - 33.7]	100 [80.5 - 100]	48.5 [40.8 - 56.3]
anti-Smith	11.4 [6.0 - 19.1]	100 [95.7 - 100]	100 [97.9 - 100]	100 [91 - 100]	5.0 [0.6 - 41]	0.9 [0.7 - 1.1]	21.4 [1.2 - 368.2]	11.4 [-6.6 - 29.4]	100 [73.5 - 100]	47.2 [39.6 - 54.8]
anti-dsDNA	33.3 [24.4 - 43.2]	97.6 [91.6 - 99.7]	96.0 [91.8 - 98.4]	100 [91 - 100]	13.8 [3.2 - 59.7]	0.7 [0.5 - 0.9]	20.2 [4.7 - 87.2]	30.9 [15.0 - 46.9]	94.6 [81.8 - 99.3]	53.6 [45.4 - 61.8]
C3	4.8 [1.6 - 10.8]	96.4 [89.8 - 99.2]	98.3 [95 - 99.6]	100 [91 - 100]	1.3 [0 - 77.9]	1.0 [0.8 - 1.2]	1.3 [0.3 - 5.7]	1.1 [-18.0 - 20.3]	62.5 [24.5 - 91.5]	44.4 [37.1 - 52]
C4	9.5 [4.7 - 16.8]	95.2 [88.1 - 98.7]	97.1 [93.4 - 99.1]	100 [91 - 100]	2.0 [0.2 - 16.9]	1.0 [0.8 - 1.2]	2.1 [0.6 - 6.9]	4.7 [-14.1 - 23.5]	71.4 [41.9 - 91.6]	45.4 [37.9 - 53.1]

All performance estimates include a 95% confidence interval (95% CI). Performance characteristics, including positive likelihood ratio (+LR), negative likelihood ratio (-LR), diagnostic odds ratio (DOR), Positive predictive value (PPV) and negative predictive value (NPV) are provided as global measures of accuracy in distinguishing SLE vs. AHV. Youden’s index (Sensitivity + Specificity – 1) is presented as a percentage reflecting the overall balance between sensitivity and specificity for SLE vs. AHV.

**Table 4:** Results of McNemar test and accompanying Cohen’s g for effect size to compare discordance between marker positivity. .

Comparison	Odds Ratio	Odds Ratio 95% CI	Adjusted p-values (Benjamini-Hochberg)	Effect Size (Cohen’s g)
Any TCell marker positive vs Any CBCAPs Positive	16.0	4.9 - 98.8	p-value < 0.001	0.9
Any TCell maker positive vs Any Conventional Marker positive	3.7	1.8 - 8.2	p-value < 0.001	0.6
TC4d vs Anti-Smith	Undefined	Undefined	p-value < 0.001	1
TC4d vs Anti-dsDNA	5.3	2.4 - 14.2	p-value < 0.001	0.7
TC4d vs TIgG	10.3	3.7 - 43.1	p-value < 0.001	0.8
TC4d vs TIgM	11	4.0 - 45.7	p-value < 0.001	0.8

Adjusted p-values according to the Benjamini-Hochberg procedure was preformed to adjust for multiple comparisons. Odds ratios and 95% confidence intervals with 95% confidence intervals (CI) are provided.

To directly compare the T Cell SLE biomarkers sensitivity with anti-dsDNA and anti-Smith, diagnostic thresholds corresponding to the 95th percentile of the ARD group (strong positive diagnostic threshold) were established for each T Cell biomarker assay (**Supplementary Table 2**). At 95% specificity for SLE vs. ARD, TC4d was 37.1% (95% CI: 27.9-47.1%) sensitive for SLE, while TIgG and TIgM had sensitivity for SLE of 25.7% (95% CI: 17.7-35.2%) and 17.1% (95% CI: 10.5-25.7%), respectively. Combining anti-dsDNA, anti-Smith and the T Cell SLE biomarkers at their strong positive thresholds yielded a combined sensitivity of 56% for SLE, with 97.6% or greater specificity for SLE vs. AHV for each T Cell biomarker (**Supplementary Figure 1**). Notably, the T Cell SLE biomarkers uniquely identified 20% of the SLE population while anti-dsDNA and anti-Smith uniquely identified 9% and 1%, respectively. Collectively, the data demonstrates that the T Cell SLE biomarkers augments sensitivity for SLE while maintaining high positive predictive value for SLE.

### T cell biomarkers vs. conventional tests: sensitivity for SLE overlap analysis

**Figure 3:**
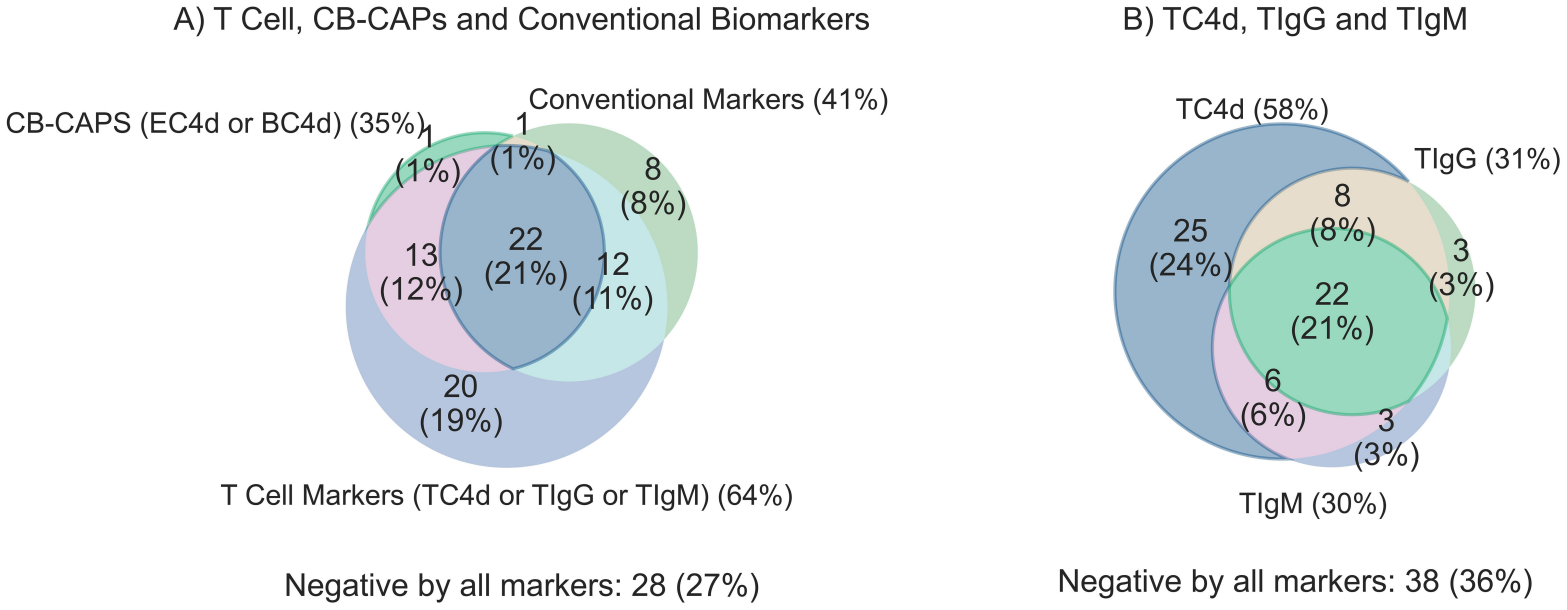
Clinical sensitivity for SLE (n=105) overlap analysis comparing T Cell SLE biomarkers (TC4d, TIgG and TIgM) to CB-CAPs (EC4d & BC4d) and conventional biomarkers (anti-dsDNA, anti-Smith and low serum complement C3 and C4). T Cell biomarkers are considered positive if values are greater than the 95th percentile of AHV, and all other biomarkers are positive based on pre-defined diagnostic thresholds. Each biomarker set is counted positive if one or more of its individual biomarker components are positive for a given subject. **(A)** Overlap analysis comparing the sensitivity for SLE among T Cell biomarkers, CB-CAPs and conventional SLE biomarkers **(B)** T Cell biomarker overlap analysis comparing the unique contributions of the individual T Cell biomarkers to the overall sensitivity of the panel.

The degree of overlap between T Cell biomarkers, EC4d, BC4d and conventional SLE tests is shown in **Figure 3A**. The combination of T Cell SLE biomarkers demonstrated significantly higher sensitivity with strong effect size for compared to conventional biomarkers (64% vs. 41%; p < 0.001, *g*=0.6), with an associated odds ratio (OR) of 3.7 (95% CI: 1.8–8.2) (**Table 4**). A similar comparison demonstrated that the T Cell biomarkers identified a greater proportion of SLE subjects compared to the combination of EC4d and BC4d (64% vs. 35%; p < 0.001). This finding, characterized by a strong effect size (*g* = 0.9), was further supported by an odds ratio of 16.0 (95% CI: 4.9–98.8). The overlap among T Cell biomarkers, depicted in **Figure 3B**, reveals that TC4d uniquely identified the largest proportion of SLE patients of any of the biomarkers, detecting 24% (25/105), while TIgG and TIgM identified 3% (3/105) and 3% (3/105), respectively. The observed discordances demonstrated statistical significance, with the odds ratio for TC4d positive and TIgG negative versus TC4d negative and TIgG positive being 10.3 (95% CI: 3.7–43.1; adjusted p < 0.001, *g* = 0.8). Similarly, for the comparison of TC4d positive and TIgM negative versus TC4d negative and TIgM positive the odds ratio was 11 (95% CI: 4.0–45.7; adjusted p < 0.001, *g* = 0.8).

**Figure 4:**
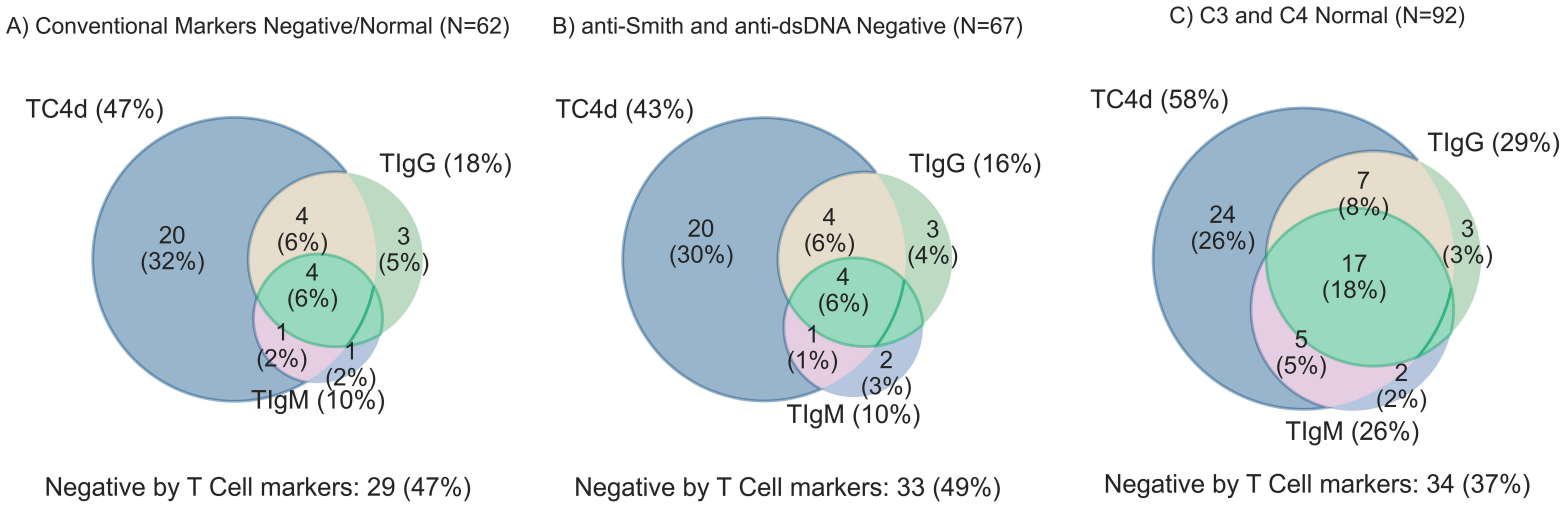
T Cell biomarker clinical sensitivity for SLE overlap analysis stratified by conventional biomarker status. T Cell biomarkers are considered positive if values are greater than the 95th percentile of AHV. **(A)** SLE subjects with all negative conventional biomarkers (anti-dsDNA, anti-Smith, and normal serum complement levels) [n=62] **(B)** anti-Smith and anti-dsDNA negative SLE (n=67) **(C)** C3 and C4 complement levels in the normal range (n=92).

The SLE cohort was stratified by conventional biomarker status to quantify the added value of T Cell biomarkers where conventional markers are negative (**Figure 4**). Notably, 33/62 (53%) of SLE patients with negative antibody profiles (anti-dsDNA and anti-Smith) and normal serum C3 and C4 (**Figure 4A**) were uniquely identified by the T Cell SLE biomarkers. In the antibody negative subset of SLE (**Figure 4B**), 34/67 (51%) had one or more T Cell biomarker positive. Among SLE patients with normal Complement levels (**Figure 4C**), 58/92 (63%) of patients tested positive for one or more of the T Cell SLE biomarkers. The conventional biomarker stratification analysis reveals the T Cell biomarkers have an approximately 50% increased relative sensitivity for SLE.

### Correlation between T Cell SLE biomarkers age and SELENA-SLEDAI 2K (SLEDAI-2K)

An anomaly analysis was performed to uncover associations between T Cell SLE biomarkers and demographic characteristics (**Supplementary Figure 2**), revealing abnormally high T Cell biomarker results were associated with younger age (52.7 [mean] ± 14.3 [SD] vs. 45.8 ± 14.7 years old). Significant elevations with small effect size in anti-Smith (Wilcoxon effect size [r] = 0.29; p = 0.03), TIgG (r = 0.27; p = 0.03), EC4d (r = 0.23; p = 0.04), and TC4d (r = 0.23; p = 0.04) were observed in SLE subjects below the median age (53 years) compared to those above it (**Supplementary Figure 4**). BC4d, TIgM, and all other conventional biomarkers did not exhibit significant differences when stratified by median age. Further stratification of SLE subjects by biomarker status (based on respective cutoffs) revealed that only anti-Smith positivity (r = 0.36; p < 0.01) and TC4d positivity (r = 0.26; p = 0.04) were significantly associated with differences in log-transformed age distributions, though both showed small effect sizes (**Supplementary Figure 5**).

Complete SLEDAI-2K data were available for 90/105 (86%) SLE subjects, primarily due to missing microscopic urinalysis data. The correlation matrix between the T Cell Lupus biomarkers and SLEDAI-2K components (**Supplementary Figure 3**) generally showed a weak correlation between the T Cell SLE biomarkers and individual SLEDAI-2K features. The T Cell SLE biomarkers were moderately correlated with one another (TC4d and TIgG: ρ = 0.49; TC4d and TIgM: ρ = 0.6; TIgG and TIgM: ρ = 0.5). TC4d, TIgG and TIgM were weakly correlated with low complement (ρ = 0.09, ρ = 0.25 and ρ = 0.23, respectively). Similarly, the correlation between TC4d, TIgG and TIgM and anti-dsDNA was generally poor (ρ = 0.42, ρ = 0.39 and ρ = 0.38, respectively). Mean clinical SLEDAI-2K scores were not significantly (p<0.05) different between any of the individual T Cell SLE biomarkers when stratified by positive or negative using the diagnostic cutoffs based on the 95^th^ percentile of AHVs.

### Leukopenia and lymphopenia lack association with the T cell SLE biomarkers

**Figure 5:**
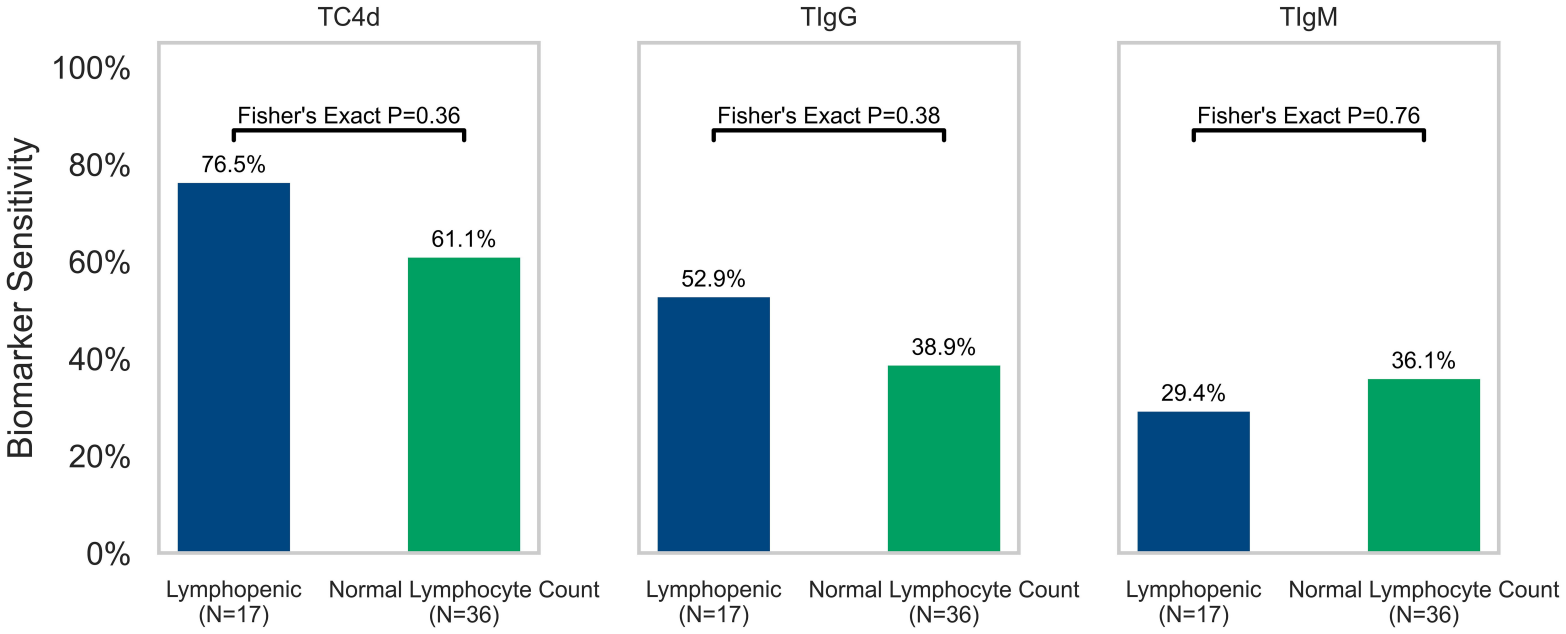
Association of T Cell SLE biomarkers (TC4d, TIgG and TIgM) with lymphopenia (defined as a lymphocyte count <1,000 cells/mm^3^) in the 50% (53/105) of SLE patients where lymphocyte count data was available for analysis. The sensitivity of each T Cell biomarker for SLE is plotted and stratified by lymphopenia status (green = normal lymphocyte count and blue = lymphopenic) based on lymphocyte counts obtained within 15 days of blood draw for T Cell biomarkers testing. Fisher’s Exact Test was performed to determine whether sensitivity of T Cell biomarkers for SLE (based on 95^th^ percentile of AHV cutoffs) was significantly associated with lymphopenia.

Consistent with the notion that abnormal T Cell SLE biomarkers do not correlate with lymphopenia (<1,000 cells/mm^3^), the positive rate of each T Cell biomarker stratified by lymphopenic vs. normal lymphocyte counts in the SLE cohort with available lymphocyte count data (within 15 days of T Cell SLE biomarker blood draw) was not significantly associated with TC4d (76.5% vs. 61.1%, p = 0.36), TIgG (52.9% vs. 38.9%, p = 0.38) or TIgM (29.4% vs. 36.1%, p = 0.76) status based on the 95^th^ percentile of AHV cutoffs (**Figure 5**). However, lymphocyte counts (cells/mm^3^) tended to be lower in TC4d-positive patients (1244.9 ± 596.49 vs. 2046.1 ± 1185.7, p = 0.02), TIgG-positive (1145.7 ± 563.7 vs. 1801.7 ± 1034.5, p = 0.02) and TIgM-positive (1265.0 ± 544.4 vs. 1646.6 ± 1041.2, p = 0.32) patients. TC4d showed a weak inverse correlation with leukopenia (ρ = -0.07), while TIgG and TIgM were weakly correlated with leukopenia (ρ = 0.29 and ρ = 0.17, respectively). While the logistic regression analysis showed a significant association between TIgG and leukopenia (p = 0.04), the Pearson correlation was low (ρ = 0.29). In contrast, TC4d showed a very weak inverse correlation with leukopenia (ρ = -0.07, p = 0.31), and TIgM demonstrated a weak positive correlation (ρ = 0.17, p = 0.21), neither of which were statistically significant.

## Discussion

The findings of this study align with evidence from other studies in the U.S. and China, supporting the generalizability of anti-lymphocyte autoantibodies and cell-bound complement activation product deposition on T Cells — TC4d, TIgG and TIgM as SLE diagnostic biomarkers with increased diagnostic sensitivity compared to conventional biomarkers. The T Cell SLE biomarkers differentiated SLE patients from apparently healthy volunteers and those with autoimmune and non-autoimmune rheumatic disorders. Comparative biomarker analyses demonstrated the T Cell SLE biomarkers and BC4d demonstrated superior diagnostic performance compared to conventional SLE biomarkers with the potential to identify an upwards of 50% of serologically inactive SLE patients. Our findings are consistent with those of Liu et al. and Huang et al., who demonstrated that TC4d, TIgG and TIgM exhibit strong diagnostic performance for SLE, including in serologically inactive patients (11, 12).

Measurement of CB-CAPs on other cell types, particularly erythrocytes (EC4d) and B lymphocytes (BC4d), has been recognized to distinguish SLE from OD and NHV more effectively than conventional SLE biomarkers (14, 30). CB-CAPs are also independently associated with SLE disease severity (33) and disease activity (29) even when adjusting for typical factors associated with severity and activity, such as age, disease duration and race/ethnicity. Prior evidence suggests TIgG and TIgM autoantibodies appear to activate complement, leading to the formation of cell-surface TC4d (11). The temporal relationship between T Cell autoantibody formation and TC4d deposition remains unclear, representing a logical next step to explore the relative abundance of these biomarkers in association with the clinical fluctuations of the disease.

Future studies should also address the potential confounding effects medications targeting T Cells and B Cells may have on these biomarkers. The present study is insufficiently powered for sub-analysis stratification by individual medications. Prior longitudinal analyses have shown a significant inverse correlation between EC4d and SLEDAI scores in highly active SLE patients receiving immunosuppressants followed prospectively (29, 34). Given the significantly greater sensitivity of T Cell biomarkers compared to EC4d and BC4d, it’s plausible that these biomarkers could prove even more effective in a sufficiently active SLE population. The development of biomarkers capable of serving as sensitive and objective measures of disease activity, severity and subtypes remains a critical unmet need (35).

The hypothesis that T Cell biomarker signatures may serve as indicators of SLE disease burden stems from evidence linking T Cell autoantibodies to the polarization of T Cells toward pro-inflammatory Th1 and Th17 subsets over regulatory T Cells (6), as well as enhanced adhesion and migratory signaling (36). T Cell autoantibodies have been shown to influence T Cell signaling, migration, and adhesion, contributing to organ-specific targeting in SLE. These findings suggest that T Cell autoantibodies are active participants in disease processes, supporting their potential as biomarkers for diagnosis and disease activity (36). The collective data indicates that T Cell autoantibodies may be participants in disease processes rather than innocent bystanders, further accentuating their potential role as biomarkers for diagnosis, disease activity and burden. Although our data did not demonstrate a significant correlation between T Cell biomarker levels and SLEDAI scores (with or without immunological components), this lack of association may be attributable to the relatively low disease activity in this well-established SLE cohort (mean SLEDAI = 3.5; median SLEDAI score = 2).

This dysregulation of T Cell activity by autoantibodies not only impacts intracellular signaling and migratory behavior but also extends to alterations in the balance of T Cell subsets. Anti-lymphocyte antibodies, for example, have long been linked to shifts in the ratio of helper (CD4) to cytotoxic (CD8) cells, potentially driven by selective clearance (37). Our findings, consistent with those of Liu et al., suggest a lack of correlation lymphopenia and abnormal levels of T Cell autoantibodies or TC4d, indicating that this functional abnormality may be obscured within the broader immune milieu. Recent evidence suggests a perturbation in a recently uncovered axis between pro-inflammatory CXCL13^+^ T peripheral helper (T_PH_) and IL-22^+^ T regulatory cells modulated by an aryl hydrocarbon (AHR) and AP-1 family protein (JUN) axis that is opposed by Type 1 interferon (38). Collectively, these data suggest that the complex interplay of T Cell subsets and their regulatory networks is central to SLE pathogenesis. Anti-lymphocyte antibodies and TC4d may both reflect and contribute to this dysregulation, positioning these biomarkers as potential diagnostic indicators, surrogates of disease activity and treatment response.

The role of T Cell autoantibodies and TC4d in disrupting T Cell subsets not only advances our understanding of SLE pathogenesis but also points to a critical need for improved diagnostic biomarkers. Delays in SLE diagnosis, often due to the limitations of current biomarkers, can lead to worsened outcomes (17). Improved diagnostic markers, such as the T Cell biomarkers described here, could help reduce these delays and improve patient care. Thus, if these T Cell biomarkers improve the ability of clinicians to differentiate between SLE, OD and patients with falsely positive ANA, the morbidity, mortality and suffering of patients with SLE may be significantly curtailed.

### Strengths and limitations

The present study bears several notable strengths. The testing cohort was developed from 5 sites across the United States, across both academic and community rheumatology clinics, ensuring a selection of SLE patients from different providers and demographic/geographic settings. The patients were sampled from the outpatient clinic setting, offering a range of disease activity that tended towards milder disease, reflecting the most common use case and environment for SLE diagnostic testing. Further, the diversity of control subjects tested, inclusive of apparently healthy individuals as well as both autoimmune and non-autoimmune rheumatic disease controls, offers a broad understanding of cell-bound complement activation and anti-lymphocyte autoantibody presence independent of SLE.

However, several limitations should be considered. The SLE cohort had established disease, meeting formal ACR classification criteria for SLE and the mean age of the cohort is older than the typical age ofonset. Therefore, further investigation of the relative diagnostic sensitivity of the T Cell biomarkers in probable or suspected SLE (subjects who fail to meet classification criteria) should be explored. However, it should be noted that CB-CAPs (EC4d and BC4d) have shown greater sensitivity in probable SLE patients compared to serum complement and specific autoantibodies (14), suggesting that TC4d, which is a by-product of similar complement activation events in particular, may exhibit similar performance. Additionally, the SLE cohort tested in this study tended to have both mild clinical activity levels and mild disease manifestations, which may underrepresent the sensitivity of these biomarkers relative to a SLE cohort with higher disease activity and more severe manifestations.

Another consideration to be accounted for in the interpretation of this analysis is the fact that the SLE cohort was treated with a significant proportion of SLE patients were taking immunosuppressants. The degree to which medications affect the T Cell biomarkers studied herein remains to be elucidated; however, the finding that the T Cell biomarkers test performance was superior to conventional biomarkers in the setting of prevalent, low dose corticosteroid use suggests robustness of biomarker applicability at the level of the medication exposure present in this cohort. Furthermore, the prevalent use of immunosuppressants may further explain the relatively low clinical disease activity present in the SLE cohort tested.

Lastly, the dataset was insufficiently powered to consider machine learning approaches which may unlock further diagnostic potential via the inter-relationships of T Cell biomarkers with existing CB-CAPs and conventional autoantibody testing. Despite these limitations, the robustness of the findings in the present study supports the need for further investigation to validate and refine the diagnostic utility of the T Cell SLE biomarkers and to explore the potential of these biomarkers to serve as objective measures of SLE disease activity.

## Conclusions

T Cell biomarkers including TC4d, TIgG and TIgM, represent a significant advancement in the diagnosis of SLE, demonstrating superior diagnostic performance compared to conventional SLE biomarkers. The ability to accurately identify SLE, even in serologically inactive patients, heightens their potential to address long-standing diagnostic delays that contribute to disease progression and irreversible organ damage. Integrating these biomarkers into diagnostic testing approaches could significantly improve early detection and treatment at crucial phases of the disease where the greatest potential lies to affect the trajectory of disease progression. Further studies of T Cell biomarker dynamics in SLE, particularly in longitudinal cohorts is warranted.

## Data Availability

The original contributions presented in the study are included in the article/**Supplementary Material**, further inquiries can be directed to the corresponding author/s.

## References

[1] Fava A Petri M. Systemic lupus erythematosus: Diagnosis and clinical management. J Autoimmun. (2019) 96:1–13. doi: 10.1016/j.jaut.2018.11.001 30448290 PMC6310637

[2] Li H Boulougoura A Endo Y Tsokos GC. Abnormalities of T cells in systemic lupus erythematosus: new insights in pathogenesis and therapeutic strategies. J Autoimmun. (2022) 132:102870. doi: 10.1016/j.jaut.2022.102870 35872102

[3] Suárez-Fueyo A Bradley SJ Tsokos GC. T cells in systemic lupus erythematosus. Curr Opin Immunol. (2016) 43:32–8. doi: 10.1016/j.coi.2016.09.001 PMC5125867 27636649

[4] Sano H Kumagai S Namiuchi S Uchiyama T Yodoi J Maeda M. Systemic lupus erythematosus sera antilymphocyte reactivity: detection of antibodies to Tac-antigen positive T cell lines. Clin Exp Immunol. (1986) 63:8–16. 3006953 PMC1577357

[5] Mimura T Fernsten P Jarjour W Winfield JB. Autoantibodies specific for different isoforms of CD45 in systemic lupus erythematosus. J Exp Med. (1990) 172:653–6. doi: 10.1084/jem.172.2.653 PMC2188347 2142723

[6] Juang YT Wang Y Solomou EE Li Y Mawrin C Tenbrock K. Systemic lupus erythematosus serum IgG increases CREM binding to the IL-2 promoter and suppresses IL-2 production through CaMKIV. J Clin Invest. (2005) 115:996–1005. doi: 10.1172/JCI22854 15841182 PMC1070410

[7] Lenert P Lenert G Senécal JL. CD4-reactive antibodies in systemic lupus erythematosus. Hum Immunol. (1996) 49:38–48. doi: 10.1016/0198-8859(96)00058-4 8839774

[8] Terasaki PI Mottironi VD Barnett EV. Cytotoxins in disease. Autocytotoxins in lupus. N Engl J Med. (1970) 283:724–8. doi: 10.1056/NEJM197010012831403 5311527

[9] Winfield JB Winchester RJ Kunkel HG. Association of cold-reactive antilymphocyte antibodies with lymphopenia in systemic lupus erythematosus. Arthritis Rheumatol. (1975) 18:587–94. doi: 10.1002/art.1780180609 1081876

[10] Takeuchi T Pang M Amano K Koide J Abe T. Reduced protein tyrosine phosphatase (PTPase) activity of CD45 on peripheral blood lymphocytes in patients with systemic lupus erythematosus (SLE). Clin Exp Immunol. (1997) 109:20–6. doi: 10.1046/j.1365-2249.1997.4371334.x PMC1904709 9218819

[11] Liu CC Manzi S Ahearn JM. Antilymphocyte autoantibodies generate T cell-C4d signatures in systemic lupus erythematosus. Transl Res. (2014) 164:496–507. doi: 10.1016/j.trsl.2014.07.007 25168018

[12] Huang JJ Mao TJ Zhang ZY Feng G. Systemic evaluation of lymphocyte-bound C4d and immunoglobulins for diagnosis and activity monitoring of systemic lupus erythematosus. Clin Biochem. (2023) 118:110600. doi: 10.1016/j.clinbiochem.2023.110600 37343744

[13] Perl A Agmon-Levin N Crispín JC Jorgensen TN. Editorial: New biomarkers for the diagnosis and treatment of systemic lupus erythematosus. Front Immunol. (2022) 13:1009038. doi: 10.3389/fimmu.2022.1009038 36311710 PMC9599399

[14] Ramsey-Goldman R Alexander RV Massarotti EM Wallace DJ Narain S Arriens C. Complement activation in patients with probable systemic lupus erythematosus and ability to predict progression to american college of rheumatology-classified systemic lupus erythematosus. Arthritis Rheumatol. (2020) 72:78–88. doi: 10.1002/art.41093 31469249 PMC6972605

[15] Bruce IN Buie J Bloch L Bae SC Costenbader K Levy RA. Lupus spectrum ambiguity has long-term negative implications for patients. Lupus Sci Med. (2023) 10:e000856. doi: 10.1136/lupus-2022-000856 37534513 PMC9835935

[16] Al Sawah S Pereira E Mariz E Pestana E. SAT0423 understanding delay in diagnosis, access to care and satisfaction with care in lupus: findings from a cross-sectional online survey in the United States. Ann Rheum Dis. (2015) 74:812–2. doi: 10.1136/annrheumdis-2015-eular.1159

[17] Kernder A Richter JG Fischer-Betz R Winkley-Rohfling B Brinks R Aringer M. Delayed diagnosis adversely affects outcome in systemic lupus erythematosus: Cross sectional analysis of the LuLa cohort. Lupus. (2021) 30:431–8. doi: 10.1177/0961203320983445 PMC7933718 33402036

[18] Kapsala NN Nikolopoulos DS Flouda SP Chavatza AP Tseronis DD Aggelakos MD. From first symptoms to diagnosis of systemic lupus erythematosus: mapping the journey of patients in an observational study. Clin Exp Rheumatol. (2023) 41:74–81. doi: 10.55563/clinexprheumatol/x3s9td 35485411

[19] Oglesby A Korves C Laliberté F Dennis G Rao S Suthoff ED. Impact of early versus late systemic lupus erythematosus diagnosis on clinical and economic outcomes. Appl Health Econ Health Policy. (2014) 12:179–90. doi: 10.1007/s40258-014-0085-x 24573911

[20] Bruce IN O’Keeffe AG Farewell V Hanly JG Manzi S Su L. Factors associated with damage accrual in patients with systemic lupus erythematosus: results from the Systemic Lupus International Collaborating Clinics (SLICC) Inception Cohort. Ann Rheum Dis. (2015) 74:1706–13. doi: 10.1136/annrheumdis-2013-205171 PMC4552899 24834926

[21] Chambers SA Allen E Rahman A Isenberg D. Damage and mortality in a group of British patients with systemic lupus erythematosus followed up for over 10 years. Rheumatol (Oxford). (2009) 48:673–5. doi: 10.1093/rheumatology/kep062 19359343

[22] Segura BT Bernstein BS McDonnell T Wincup C Ripoll VM Giles I. Damage accrual and mortality over long-term follow-up in 300 patients with systemic lupus erythematosus in a multi-ethnic British cohort. Rheumatol (Oxford). (2020) 59:524–33. doi: 10.1093/rheumatology/kez516 PMC8414923 31377781

[23] Urowitz MB Gladman DD Ibañez D Fortin PR Bae SC Gordon C. Evolution of disease burden over five years in a multicenter inception systemic lupus erythematosus cohort. Arthritis Care Res (Hoboken). (2012) 64:132–7. doi: 10.1002/acr.20648 21954226

[24] Altabás-González I Rua-Figueroa I Mouriño C Roberts K Jimenez N Martinez-Barrio J. Damage in a large systemic lupus erythematosus cohort from the Spanish Society of Rheumatology Lupus Registry (RELESSER) with emphasis on the cardiovascular system: a longitudinal analysis. Lupus Sci Med. (2024) 11:e001064. doi: 10.1136/lupus-2023-001064 39097409 PMC11331961

[25] Aringer M Costenbader K Daikh D Brinks R Mosca M Ramsey-Goldman R. 2019 European league against rheumatism/American college of rheumatology classification criteria for systemic lupus erythematosus. Arthritis Rheumatol. (2019) 71:1400–12. doi: 10.1002/art.40930 PMC6827566 31385462

[26] Satoh M Chan EK Ho LA Rose KM Parks CG Cohn RD. Prevalence and sociodemographic correlates of antinuclear antibodies in the United States. Arthritis Rheumatol. (2012) 64:2319–23. doi: 10.1002/art.34380 PMC3330150 22237992

[27] Hanly JG Thompson K McCurdy G Fougere L Theriault C Wilton K. Measurement of autoantibodies using multiplex methodology in patients with systemic lupus erythematosus. J Immunol Methods. (2010) 352:147–52. doi: 10.1016/j.jim.2009.10.003 19836394

[28] Meroni PL Schur PH. ANA screening: an old test with new recommendations. Ann Rheum Dis. (2010) 69:1420–2. doi: 10.1136/ard.2009.127100 20511607

[29] Merrill JT Petri MA Buyon J Buyon J Ramsey-Goldman R Kalunian K. Erythrocyte-bound C4d in combination with complement and autoantibody status for the monitoring of SLE. Lupus Sci Med. (2018) 5:e000263. doi: 10.1136/lupus-2018-000263 29868177 PMC5976122

[30] Putterman C Furie R Ramsey-Goldman R Askanase A Buyon J Kalunian K. Cell-bound complement activation products in systemic lupus erythematosus: comparison with anti-double-stranded DNA and standard complement measurements. Lupus Sci Med. (2014) 1:e000056. doi: 10.1136/lupus-2014-000056 25396070 PMC4225732

[31] Dervieux T Conklin J Ligayon JA Wolover L T Alexander RV. Validation of a multi-analyte panel with cell-bound complement activation products for systemic lupus erythematosus. J Immunol Methods. (2017) 446:54–9. doi: 10.1016/j.jim.2017.04.001 28389175

[32] O’Malley T Xie F Su Y Zack D Haechung C Grabner M. Complement activation products vs standard ANA testing: Treatment outcomes, diagnosis, and economic impact (CAPSTONE) in systemic lupus erythematosus. J Manag Care Spec Pharm. (2022) 28:1021–32. doi: 10.18553/jmcp.2022.22039 PMC12101568 35775579

[33] Arriens C Alexander RV Narain S Saxena A Collins CE Wallace DJ. Cell-bound complement activation products associate with lupus severity in SLE. Lupus Sci Med. (2020) 7:e000377. doi: 10.1136/lupus-2019-000377 32371480 PMC7228655

[34] Buyon J Furie R Putterman C Ramsey-Goldman R Kalunian K Barken D. Reduction in erythrocyte-bound complement activation products and titers of anti-C1q antibodies associate with clinical improvement in systemic lupus erythematosus. Lupus Sci Med. (2016) 3:e000165. doi: 10.1136/lupus-2016-000165 27752336 PMC5051407

[35] Lateef A Petri M. Unmet medical needs in systemic lupus erythematosus. Arthritis Res Ther. (2012) 14 Suppl 4:S4. doi: 10.1186/ar3919 23281889 PMC3535719

[36] Li Y Harada T Juang YT Wang Y Zidanic M Tung K. Phosphorylated ERM is responsible for increased T cell polarization, adhesion, and migration in patients with systemic lupus erythematosus. J Immunol. (2007) 178:1938–47. doi: 10.4049/jimmunol.178.3.1938 17237445

[37] Winfield JB Shaw M Yamada A Minota S. Subset specificity of antilymhocyte antibodies in systemic lupus erythematosus. II. Preferential reactivity with T4 + cells is associated with relative depletion of autologous T4 + cells. Arthritis Rheumatol. (1987) 30:162–8. doi: 10.1002/art.1780300206 2950862

[38] Law C Wacleche VS Cao Y Pillai A Sowerby J Hancock B. Interferon subverts an AHR-JUN axis to promote CXCL13+ T cells in lupus. Nature. (2024) 631:857–66. doi: 10.1038/s41586-024-07627-2 PMC11628166 38987586

