# Supplementary material for: Multi-centered clinical validation demonstrating superior precision in lupus diagnosis: T cell autoantibodies and TC4d outperform conventional lupus erythematosus biomarkers
Source: Front Immunol. 2025 Feb 26;16:1518208. doi: 10.3389/fimmu.2025.1518208 (PMC11907206; doi:10.3389/fimmu.2025.1518208)
Supplement: Supplementary file 1 [file DataSheet1.docx]

**Supplemental Materials**

**Detailed TC4d and T Cell autoantibody assays (TC4d, TIgG and TIgM)**

TC4d and T-Cell autoantibodies (TIgG and TIgM) quantification - Following erythrocyte lysis from whole blood (400µl) using an ammonium chloride-based reagent (BD Pharm Lyse, BD Biosciences) and centrifugation (5 minutes @ 2600 RPM), cell pellet is re-suspended in 135µl of a 4% normal bovine serum solution. A 25µl aliquot is incubated with the target antibody cocktail for 20 minutes at room temperature in dark conditions. The target antibody cocktail comprises of biotinylated anti-C4d antibody (mouse anti-human C4d, Quidel Inc.); anti-IgG antibody conjugated to Alexa Fluor 700; anti-IgM antibody conjugated to PE; anti-CD3 antibody conjugated to Pacific Blue and anti-CD19 antibody conjugated to APC-Cy7. A second aliquot is incubated with the isotype cocktail, comprising of biotinylated non-specific mouse IgG1 kappa antibody (MOPC-21, BD Biosciences); Mouse isotype conjugated to PE; mouse isotype conjugated to Alexa Fluor 700; anti-CD3 antibody conjugated to Pacific Blue and anti-CD19 antibody conjugated to APC-Cy7. After washing, cells are incubated with streptavidin conjugated to Alexa Fluor for 20 minutes at room temperature in the dark. A total of 100,000 events are collected.

All analyses were done with BD FACS Lyric (3 laser, 12 color configuration) equipped with BD FACSuite software (v1.5.0.923). FACS data analysis was performing using FCSExpress RUO Software version 7.10.0007 [64-bit] (DeNovo Software, Pasadena, CA).

**Supplementary Table 1:** Other ENA autoantibody performance characteristics using previously established diagnostic thresholds from IVD cleared enzyme-linked fluorescent assays (ELFA).

|  | **Sensitivity %**  **[95% CI]** | **Specificity %**  **[95% CI]** | | | **SLE vs AHV** | | | | | | |
| --- | --- | --- | --- | --- | --- | --- | --- | --- | --- | --- | --- |
| **Biomarker** | **SLE** | **AHV** | **ARD** | **OD** | **+LR**  **[95% CI]** | **-LR**  **[95% CI]** | **OR**  **[95% CI]** | **Youden %**  **[95% CI]** | **PPV %**  **[95% CI]** | **NPV %**  **[95% CI]** |  |
| Anti-Ro60 | 41  [31.5 - 51] | 97.6  [91.6 - 99.7] | 86.9  [81.1 - 91.4] | 97.4  [86.5 - 99.9] | 17.0  [4.1 - 71.1] | 0.6  [0.5 - 0.8] | 28.1  [6.6 - 120.4] | 38.5  [23.5 - 53.6] | 95.6  [84.9 - 99.5] | 56.6  [48.1 - 64.9] |  |
| Anti-Ro52 | 23.8  [16.0 - 33.1] | 100  [95.7 - 100] | 90.2  [84.9 - 94.1] | 100  [91 - 100] | 10.4  [2.2 - 49.7] | 0.8  [0.6 – 1.0] | 51.9  [3.1 - 867] | 23.8  [7.1 - 40.5] | 100  [86.3 - 100] | 50.9  [43 - 58.8] |  |
| Anti-U1RNP | 20.0  [12.8 - 28.9] | 100  [95.7 - 100] | 98.9  [96.1 - 99.9] | 100  [91 - 100] | 8.7  [1.7 - 45] | 0.8  [0.7 – 1.0] | 41.5  [2.5 - 696.9] | 20.0  [2.9 - 37.1] | 100  [83.9 - 100] | 49.7  [41.9 - 57.5] |  |
| Anti-RNP70 | 13.3  [6.8 – 19.8] | 100  [95.7 – 100] | 99.4  [96.8 – 99.9] | 100  [91 – 100] | 5.3  [0.7 – 40.6] | 0.9  [0.6 – 1.3] | 6.0  [0.8 – 47.2] | 13.3  [6.8 – 19.8] | 100  [65.1 – 100] | 52.9  [44.4 – 61.5] |  |
| Anti-C1q | 14.3  [7.6 – 21.0] | 91.6  [85.6 – 97.5] | 94.2  [89.7 – 96.8] | 92.3  [79.7 – 97.4] | 1.9  [0.5 – 6.4] | 0.9  [0.6 – 1.4] | 2.0  [0.6 – 7.3] | 6.6  [0 – 17.3] | 65  [42.5 – 61.2] | 0.52  [42.5 – 61.2] |  |

**Supplementary Table 2.** Performance Characteristics of T Cell Biomarkers (based on the 95^th^ percentile of ARD group as the reference interval threshold), CB-CAPs and conventional SLE biomarkers (based on previously established diagnostic thresholds). All performance estimates include a 95% confidence interval (95% CI). Performance characteristics, including positive likelihood ratio (+LR), negative likelihood ratio (-LR), diagnostic odds ratio (DOR), Positive predictive value (PPV) and negative predictive value (NPV) are provided as global measures of accuracy in distinguishing SLE vs. AHV. Youden’s index (Sensitivity + Specificity – 1) is presented as a percentage reflecting the overall balance between sensitivity and specificity for SLE vs. AHV. In cases, where specificity equals 100%, a specificity value of 97.5% was used to facilitate calculations, including +LR, -LR and DOR.

|  | Sensitivity for SLE (%) [95% CI] | Specificity for SLE (%)  [95% CI] | | | SLE vs AHV | | | | | |
| --- | --- | --- | --- | --- | --- | --- | --- | --- | --- | --- |
| **Biomarker** | **SLE** | **AHV** | **ARD** | **OD** | **+LR**  **[95% CI]** | **-LR**  **[95% CI]** | **OR**  **[95% CI]** | **Youden %**  **[95% CI]** | **PPV %**  **[95% CI]** | **NPV %**  **[95% CI]** |
| TC4d | 37.1  [27.9 - 47.1] | 97.6  [91.6 - 99.7] | 94.5  [90.1 - 97.3] | 100  [91 - 100] | 15.4  [3.6 - 65.4] | 0.6  [0.5 - 0.8] | 23.9  [5.6 - 102.8] | 34.7  [19.2 - 50.3] | 95.1  [83.5 - 99.4] | 55.1  [46.7 - 63.3] |
| TIgG | 25.7  [17.7 - 35.2] | 98.8  [93.5 - 100] | 95.1  [90.8 - 97.7] | 100  [91 - 100] | 21.3  [2.7 - 167.8] | 0.8  [0.6 - 0.9] | 28.4  [3.8 - 214] | 24.5  [7.9 - 41.2] | 96.4  [81.7 - 99.9] | 51.2  [43.2 - 59.2] |
| TIgM | 17.1  [10.5 - 25.7] | 98.8  [93.5 - 100] | 94.5  [90.1 - 97.3] | 100  [91 - 100] | 14.2  [1.6 - 129.4] | 0.8  [0.7 - 1] | 17  [2.2 - 130] | 15.9  [-1.6 - 33.5] | 94.7  [74 - 99.9] | 48.5  [40.8 - 56.3] |
| BC4d | 31.4  [22.7 - 41.2] | 100  [95.7 - 100] | 94.5  [90.1 - 97.3] | 100  [91 - 100] | 13.7  [3.1 - 60.7] | 0.7  [0.6 - 0.9] | 76.1  [4.6 - 1264.4] | 31.4  [15.6 - 47.3] | 100  [89.4 - 100] | 53.5  [45.4 - 61.6] |
| EC4d | 16.2  [9.7 - 24.7] | 100  [95.7 - 100] | 99.5  [97 - 100] | 100  [91 - 100] | 7.0  [1.2 - 41.4] | 0.9  [0.7 - 1.1] | 32.1  [1.9 - 542.5] | 16.2  [-1.3 - 33.7] | 100  [80.5 - 100] | 48.5  [40.8 - 56.3] |
| anti-Smith | 11.4  [6 - 19.1] | 100  [95.7 - 100] | 100  [98 - 100] | 100  [91 - 100] | 5.0  [0.6 - 41] | 0.9  [0.7 - 1.1] | 21.4  [1.2 - 368.2] | 11.4  [-6.6 - 29.4] | 100  [73.5 - 100] | 47.2  [39.6 - 54.8] |
| anti-dsDNA | 33.3  [24.4 - 43.2] | 97.6  [91.6 - 99.7] | 95.6  [91.5 - 98.1] | 100  [91 - 100] | 13.8  [3.2 - 59.7] | 0.7  [0.5 – 0.9] | 20.2  [4.7 – 87.2] | 30.9  [15 – 46.9] | 94.6  [81.8 – 99.3] | 53.6  [45.4 – 61.8] |
| C3 | 4.8  [1.6 – 10.8] | 96.4  [89.8 – 99.2] | 97.8  [94.5 – 99.4] | 100  [91 – 100] | 1.3  [0 – 77.9] | 1.0  [0.8 - 1.2] | 1.3  [0.3 - 5.7] | 1.1  [-18 - 20.3] | 62.5  [24.5 - 91.5] | 44.4  [37.1 - 52] |
| C4 | 9.5  [4.7 - 16.8] | 95.2  [88.1 - 98.7] | 97.3  [93.7 - 99.1] | 100  [91 - 100] | 2.0  [0.2 - 16.9] | 1.0  [0.8 - 1.2] | 2.1  [0.6 - 6.9] | 4.7  [-14.1 - 23.5] | 71.4  [41.9 - 91.6] | 45.4  [37.9 - 53.1] |


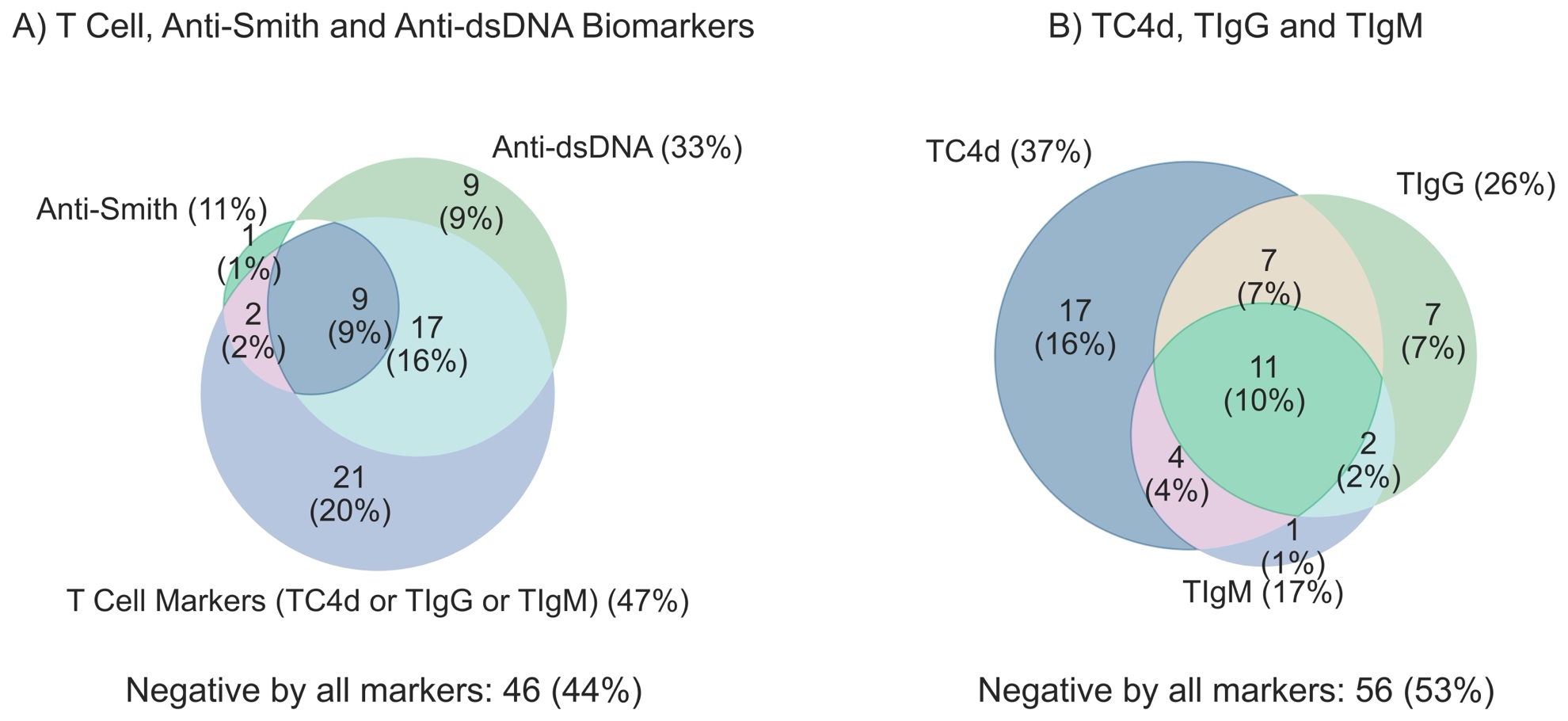


Supplementary Figure 1. Clinical sensitivity for SLE (n=105) overlap analysis comparing T Cell SLE biomarkers (TC4d, TIgG and TIgM) to conventional autoantibodies (anti-dsDNA, anti-Smith). T Cell biomarkers are considered strong positive if values are greater than the 95^th^ percentile of ARD, and all other biomarkers are positive based on pre-defined diagnostic thresholds. (A) Overlap analysis comparing the sensitivity for SLE among T Cell biomarkers, and conventional SLE biomarkers. (B) T Cell biomarker overlap analysis comparing the unique contributions of the individual T Cell biomarkers to the overall sensitivity of the panel.


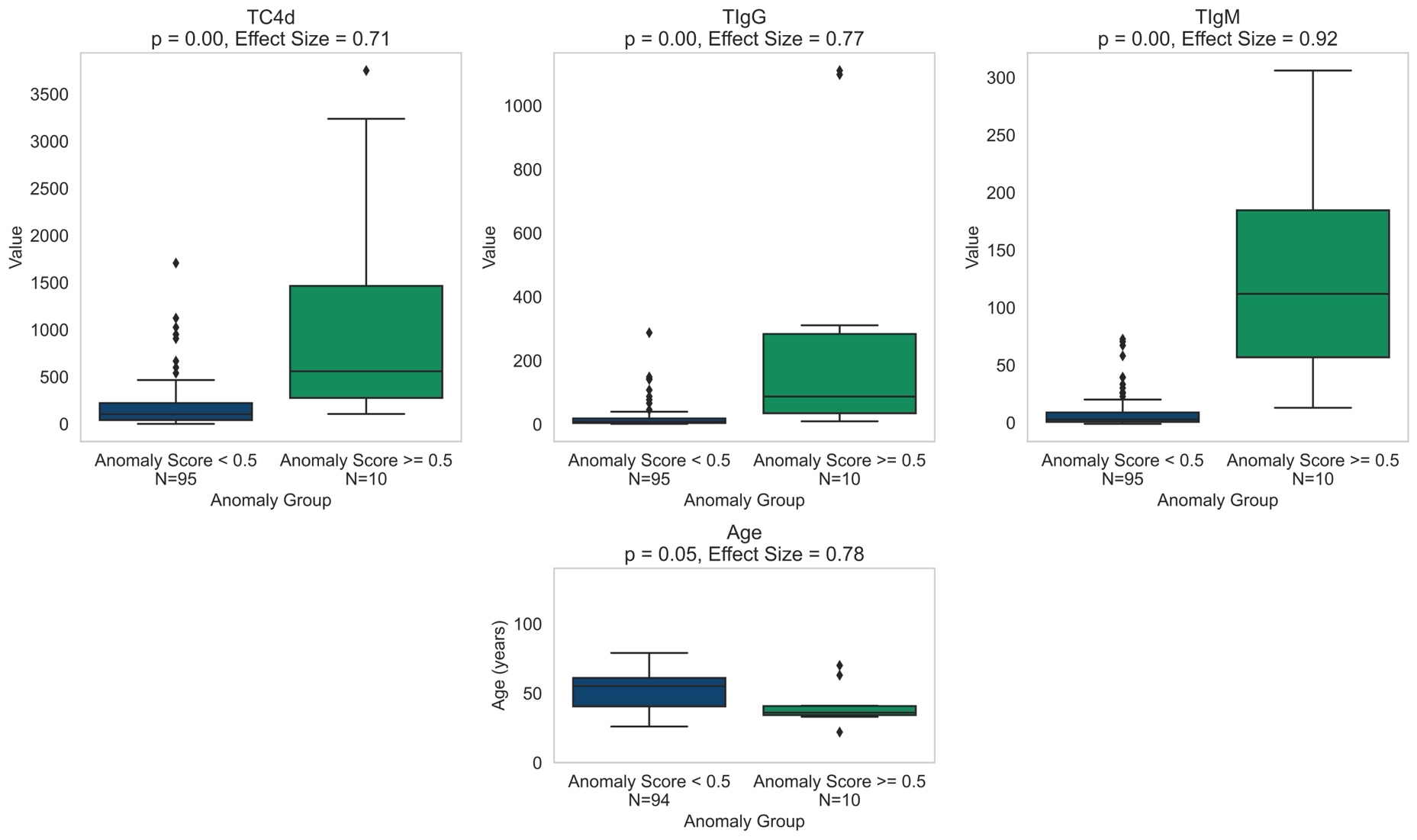


Supplementary Figure 2. The synthetic AUC for the Isolation Forest Anomaly Detection with Calibration model for the three T cell biomarkers was 0.65 for 10-fold cross-validation and 0.64 for the test set. After dividing the SLE cohort based on an anomaly score cutoff of 0.5, 31 samples were categorized as outliers (anomaly score ≥ 0.5), while 378 samples had an anomaly score of less than 0.5. The p-value of the Mann-Whitney U test for TC4d, TIgG, and TIgM between these two groups was p < 0.001, with rank biserial correlations of 0.71 for TC4d, 0.77 for TIgG, and 0.92 for TIgM. Additionally, the p-value of the t-test for age between these groups was 0.05, with a Cohen's d effect size of 0.78. The group with an anomaly score of less than 0.5 had a mean age of 51.8 years (standard deviation = 13.1), while the group with an anomaly score of 0.5 or greater had a mean age of 41.0 years (standard deviation = 14.5).


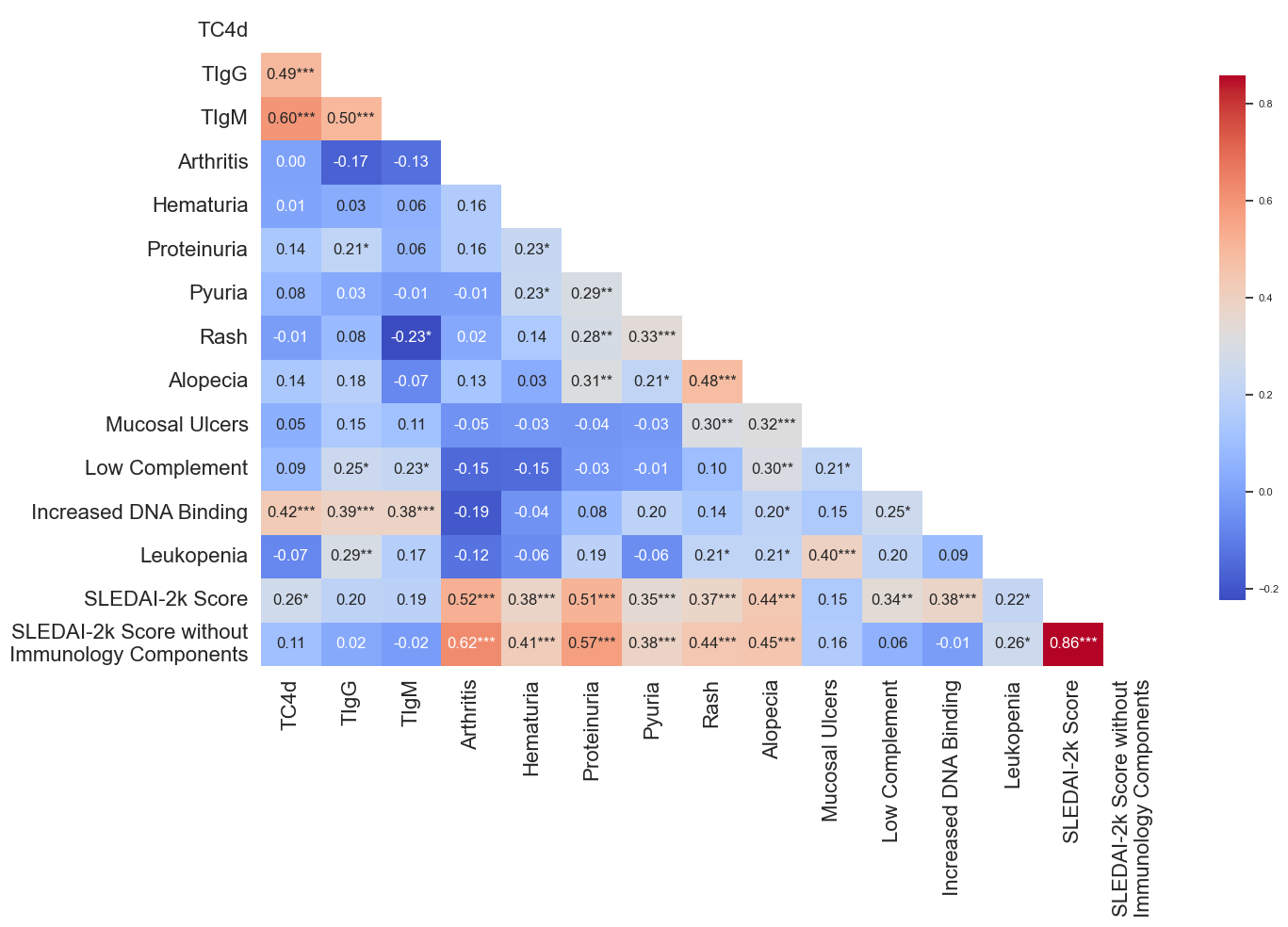


Supplementary Figure 3. T Cell SLE Biomarker correlation with SLEDAI clinical and laboratory features. Complete SLEDAI 2k information was available for 90 subjects and one additional subject had all clinical criteria but lacked immunological components; therefore, the analysis for SLEDAI score without immunological components consisted of 91 subjects. . Spearman rank correlation coefficient values are presented with strong positive correlation represented by darker red shades and strong inverse correlation represented by darker blue shades. Statistical significance is indicated as follows: p < 0.05 (*), p < 0.01 (**), and p < 0.001 (***).


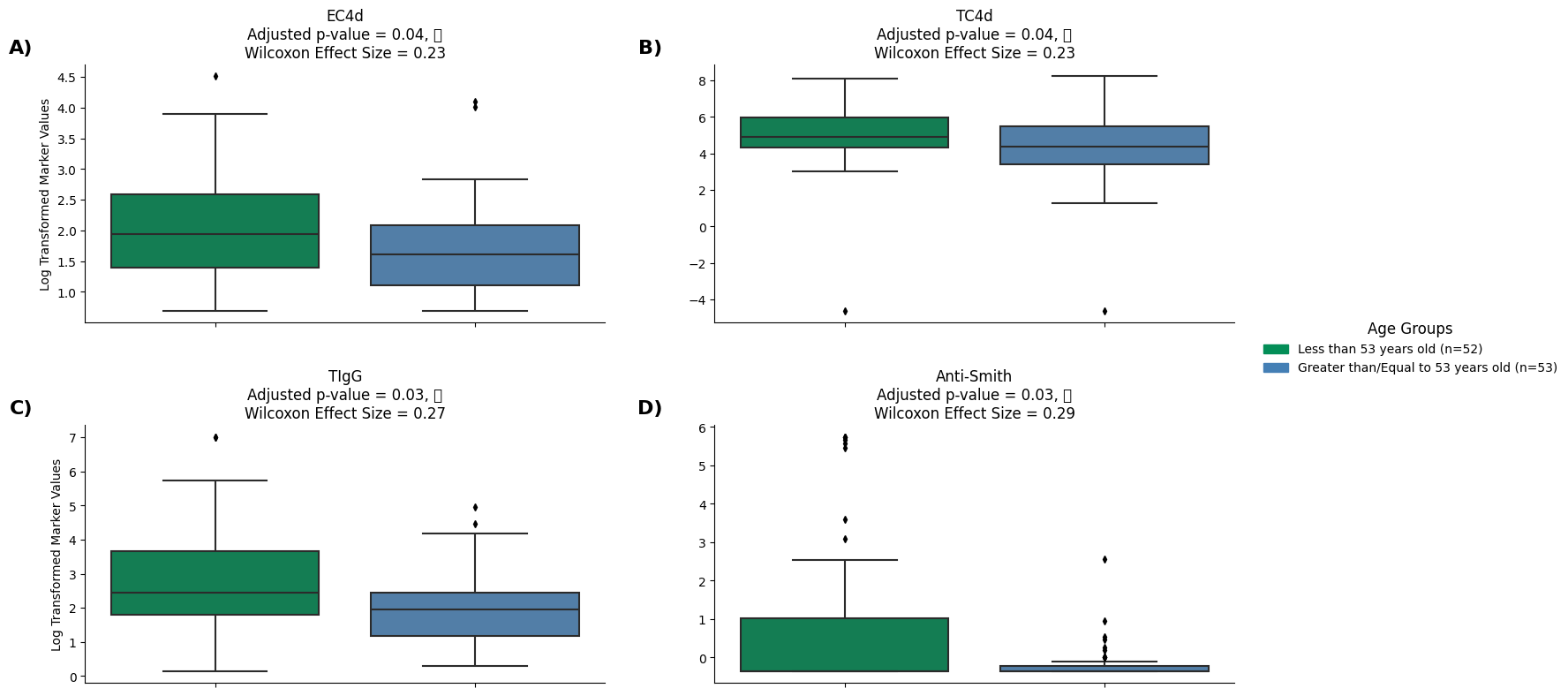


Supplementary Figure 4. Log Transformed marker values between age groups defined by the median age (53 years old) of the SLE cohort. Statistically significant differences were observed in the medians of these log-transformed markers using Mann-Whitney U-test and Wilcoxon Rank Sum test for effect size, although the effect sizes were small. Those younger than the median age tended to have higher median marker values than those older than the median age. In contrast, the median values of the other markers included in the analysis did not show significant differences between the age groups.


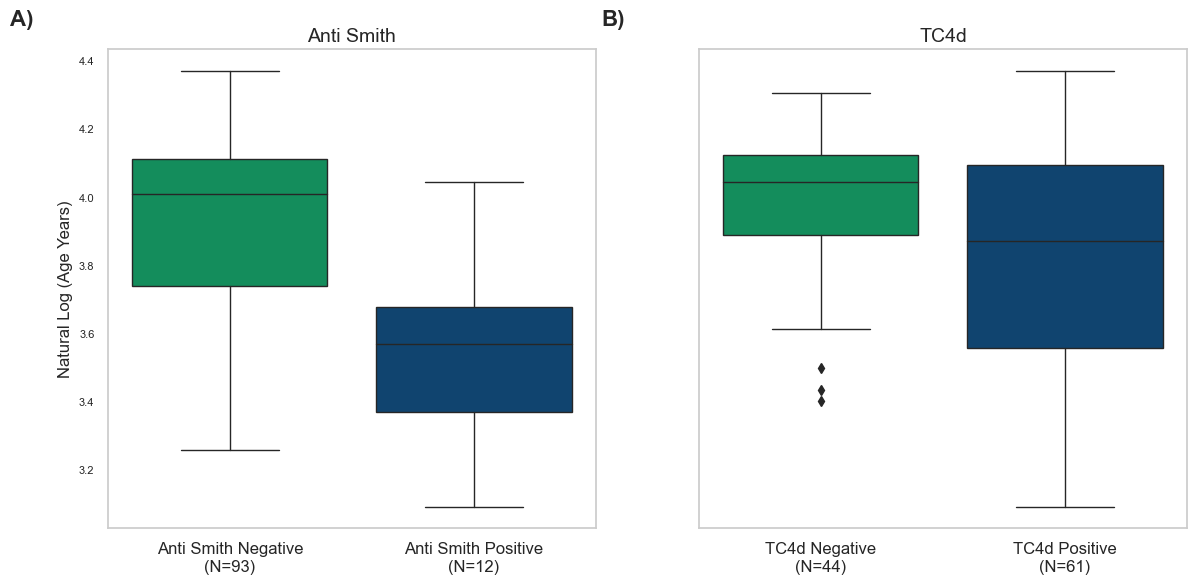


Supplementary Figure 5. Log-transformed age was analyzed in relation to marker positivity. Statistically significant differences in the median log-transformed age by marker positivity were found using the Mann-Whitney U-test, with effect sizes assessed by the Wilcoxon Rank Sum test, although the effect sizes were small. Subjects who were negative for these markers tended to have higher log-transformed ages. In contrast, no statistically significant differences were observed in the median log-transformed ages for the other markers.
